# Differences in phyllosphere microbiomes among different *Populus* spp. in the same habitat

**DOI:** 10.3389/fpls.2023.1143878

**Published:** 2023-03-30

**Authors:** Jiaying Liu, Weixi Zhang, Yuting Liu, Wenxu Zhu, Zhengsai Yuan, Xiaohua Su, Changjun Ding

**Affiliations:** ^1^ College of Forestry, Shenyang Agriculture University, Shenyang, China; ^2^ State Key Laboratory of Tree Genetics and Breeding, Research Institute of Forestry, Chinese Academy of Forestry, Beijing, China; ^3^ Key Laboratory of Tree Breeding and Cultivation of State Forestry Administration, Research Institute of Forestry, Chinese Academy of Forestry, Beijing, China; ^4^ Research Station of Liaohe-River Plain Forest Ecosystem, Chinese Forest Ecosystem Research Network (CFERN), College of Forestry, Shenyang Agricultural University, Tieling, China

**Keywords:** phyllosphere microorganism, microbiomes, *Populus* spp., phyllosphere microbial community, phyllosphere

## Abstract

**Introduction:**

The above-ground parts of terrestrial plants are collectively known as the phyllosphere. The surface of the leaf blade is a unique and extensive habitat for microbial communities. Phyllosphere bacteria are the second most closely associated microbial group with plants after fungi and viruses, and are the most abundant, occupying a dominant position in the phyllosphere microbial community. Host species are a major factor influencing the community diversity and structure of phyllosphere microorganisms.

**Methods:**

In this study, six Populus spp. were selected for study under the same site conditions and their phyllosphere bacterial community DNA fragments were paired-end sequenced using 16S ribosomal RNA (rRNA) gene amplicon sequencing. Based on the distribution of the amplicon sequence variants (ASVs), we assessed the alpha-diversity level of each sample and further measured the differences in species abundance composition among the samples, and predicted the metabolic function of the community based on the gene sequencing results.

**Results:**

The results revealed that different Populus spp. under the same stand conditions resulted in different phyllosphere bacterial communities. The bacterial community structure was mainly affected by the carbon and soluble sugar content of the leaves, and the leaf nitrogen, phosphorus and carbon/nitrogen were the main factors affecting the relative abundance of phyllosphere bacteria.

**Discussion:**

Previous studies have shown that a large proportion of the variation in the composition of phyllosphere microbial communities was explained by the hosts themselves. In contrast, leaf-borne nutrients were an available resource for bacteria living on the leaf surface, thus influencing the community structure of phyllosphere bacteria. These were similar to the conclusions obtained in this study. This study provides theoretical support for the study of the composition and structure of phyllosphere bacterial communities in woody plants and the factors influencing them.

## Introduction

Microorganisms are an important part of the ecosystem and play an important role in its stability. Their survival and reproduction affect the healthy growth of the plants. The plants are one of the most important habitats for microorganisms, and both the underground roots and above-ground branches and leaves are colonized by a large number of microorganisms, with the above-ground environment formed by the branches and leaves called the phyllosphere (Ruinen, 1956; Vorholt, 2012; Vacher et al., 2017). For a long time, research on phyllosphere microbial communities has lagged far behind that on rhizosphere microbial communities (Lindow and Brandl, 2003; Miura et al., 2019). Compared to soil and rhizosphere, phyllosphere microorganisms are less diverse but still play a key role (Xu et al., 2022). In general, the phyllosphere refers mainly to the environment in which the leaf is formed, and the microorganisms that colonize the leaf are called phyllosphere microorganisms (Lindow and Brandl, 2003; Vorholt, 2012). The phyllosphere contains both the leaf surface and the internal leaf environment, and accordingly, phyllosphere microorganisms also include epiphytic bacteria on the leaf surface and endophytic bacteria in the leaf interior (Peñuelas and Terradas, 2014; Tkacz et al., 2020; Gong and Xin, 2021).

The total surface area of the above-ground portion of plants worldwide is estimated to be about 10^9^ km^2^ (mostly leaf surface) (Vorholt, 2012), an area about twice the size of the Earth, making it one of the largest microhabitats on the planet (Vorholt, 2012). Also, in the phyllosphere grows a large number of microorganisms, including bacteria, yeasts, filamentous fungi, archaea and algae (Lindow and Brandl, 2003; Berlec, 2012). Bacteria are the most abundant group closely associated with plants (Bringel and Couée, 2015; Bao et al., 2020), with an average of 10^6^ ~10^7^ bacterial cells per square centimeter of leaf surface, occupying a major position in the phyllosphere microbial community (Vorholt, 2012). Also, several studies show that bacteria can have very intimate interactions with plants that involve intracellular colonization and even endosymbiosis (Hardoim et al., 2015; Koskimäki et al., 2015). Much of the research on phyllosphere microbes has been focused on phyllosphere bacteria (Dickinson et al., 1975; Bodenhausen et al., 2013).

Phyllosphere microorganisms are normally attached to the surface of leaves and form complex parasitic, mutualistic relationships with host plants and other microorganisms, but most phyllosphere microorganisms are in a symbiotic relationship with their host plants (Kishore et al., 2005). Phyllosphere microorganisms are complexly diverse, with different species having different microbial communities (Leveau, 2015). It has been shown that the presence of phyllosphere microorganisms is the result of a combination of colony competition, climatic selection and host selection. The composition of phyllosphere microorganisms can vary across plants, and factors associated with the population structure of phyllosphere microorganisms include plant phenotypic traits, leaf height and position, and leaf age (Hunter et al., 2010). Environmental factors, plant genotypes and the shape of plant species all affect the community composition of phyllosphere microorganisms to varying degrees. Vokou et al. (2012); Redford et al. (2010) and Vogel et al. (2020) suggest that plant species are a major factor influencing the composition and diversity of phyllosphere microorganism communities, and that different plant species have corresponding phyllosphere microorganism communities. In contrast, Laforest-Lapointe et al. (2016) explored the different drivers influencing the composition of phyllosphere bacterial communities in trees and found that host species accounted for 27% of the factors influencing the composition of phyllosphere bacteria. Existing studies have shown that host species are a major factor influencing the community diversity and structure of phyllosphere microorganisms. The main factor of host species is related to differences in the physicochemical properties of plant leaves (Whipps et al., 2008). Different plant leaf characteristics such as stomata, trichomes, leaf thickness, nutrient content (carbon, phosphorus and soluble sugar content) and water content all influence the colonisation of phyllosphere microorganisms (Bunster et al., 1989; Yadav et al., 2005; Beattie, 2011; Kembel and Mueller, 2014; Kembel et al., 2014).

A study of the model plant *Arabidopsis thaliana* using genome wide association study (GWAS) found that different species of *A. thaliana* phyllosphere microorganisms have different community composition (Horton et al., 2014). In addition, different species of the same plant species exhibited different phyllosphere microorganisms’ communities among themselves. This has been confirmed by many scholarly studies on common cash crops such as *Solanum tuberosum*, *Capsicum frutescens* var. *grossum*, *Lycopersicon esculentum*, and *Gossypium* spp. (Adams and Kloepper, 2002; Sessitsch et al., 2002; Rasche et al., 2006a; Rasche et al., 2006b; Correa et al., 2007; Hunter et al., 2015). Hunter et al. (2010) also found that different varieties of *Lactuca sativa* had different phyllosphere bacterial communities, and that differences in bacterial community structure were related to the characteristics of the leaves themselves, such as leaf morphology and soluble carbohydrates, calcium and phenolic compounds. *Populus* spp. are the model species of choice for woody plant research because of its compact genome composition, species richness, wide distribution, ease of genetic transformation and ease of asexual reproduction (Bradshaw et al., 2000; Taylor, 2002; Beckers et al., 2016; Beckers et al., 2017; Brunner et al., 2004). The poplar germplasm conservation trial forest in Tongzhou, Beijing has six species of *Populus* spp. planted in 2015 under the same stand conditions. High-throughput sequencing analysis has become one of the most commonly used methods for analyzing the composition of phyllosphere microbial communities (Rastogi et al., 2012), increasing the extent of our knowledge of the composition of phyllosphere microbial communities. Based on this, we hypothesized: do different *Populus* spp. under the same stand conditions also have different phyllosphere bacterial communities? Are the differences in bacterial community composition and structure related to the properties of the leaves themselves, such as carbon, nitrogen, phosphorus and non-structural carbohydrates in the leaves?

## Method and materials

### Plant environmental factor analysis of different samples

The sampling site of this experiment was the International Seed Technology Park in Beixindian Village, Yujiawu Township, Tongzhou District, Beijing (116°40’46″E, 39°42’59″N). The location has a temperate continental monsoon climate, which is influenced by both winter and summer winds, resulting in a windy and arid climate in spring, more rain and higher temperatures in summer, crisp climate in autumn and cold weather in winter. The average annual temperature can reach 11.3°C, and the average precipitation is about 620mm. the terrain is flat. The poplar germplasm conservation test forest in Tongzhou, Beijing, was planted in March 2015. The test species were *Populus* × *euramaricana* ‘Bofeng 3 hao’ (YA), *P. deltoides* ‘Shanghaiguan’ × *P. deltides* ‘Harvard’ (YB), *P. nigra* ‘N46’ (YC), *P. nigra* ‘N102’ (YD), *P. × euramericana* ‘Guariento’ (YE), and *P. alba × P. glandulosa* ‘84k’ (YF). Each species was planted in a 30×30 m sample plot.

### Sample collection and processing

In August 2022, three 10 × 10 m sample plots were set up in each sample area, and five poplars of similar growth were selected in each sample plot according to the five-point sampling method. Plant samples were collected from the middle tip of the canopy in three directions (120°), and mixed into one replicate in each sample plot. Each leaf sample was cut with a pair of sterilized scissors and placed in a sterile sampling bag placed on an ice box. In order to standardize conditions as much as possible, only green, healthy, whole leaves were selected. A total of 18 samples (3 replicates × 6 tree species) were collected (**Supplementary Figure 1**). Plants were planted in each plot with the density of 2 m × 2 m. All plant samples were collected on the same day and transported to the laboratory for subsequent analysis.

In each leaf replicate, 30 g of leaf samples were placed in a 1000 mL sterile conical flask and then filled with 500 mL of sterile PBS buffer (pH 7.4, 1 × phosphate buffer). To wash the microbial cells from the leaves, sonication was performed in an ultrasonic clearing bath at a frequency of 40 kHz for 6 min, with oscillation at 200 r/min for 20 min at 30°C, followed by sonication (frequency 40 kHz) for 3 min. The microbial cells were separated from the leaves by filtering the cell suspension through a sterile nylon membrane of 0.22 μm × 50 mm. The membrane samples were stored at -80°C.

Afterwards, the plant leaves were repeatedly rinsed with sterile distilled water and the surface dried with phosphate-free filter paper. After baking at 105°C for 30 min, with drying at 65°C for more than 48 h until the samples were of constant weight. The petioles and veins were cut off, ground and passed through a 100-mesh sieve for the determination of physicochemical properties.

### Leaves chemical properties determination

Dried plant samples of 3.5-4.2 mg were weighed and sealed in a tin container and then the carbon and nitrogen contents of the leaves were determined by the elemental analyzer (Elementar Vario EL III Germany). Phosphorus of leaves was determined by the molybdenum antimony anti-colorimetric method (Li et al., 2019). The content of soluble sugars and starch in the leaves was determined by the anthrone colorimetric method (Du et al., 2020).

### DNA extraction and high-throughput sequencing

All DNA was extracted using the Fast^®^DNA SPIN kit (MP Biomedicals, Santa Ana, CA, USA) according to the manufacturer’s instructions. The amount and quality of DNA was measured by NanoDrop NC2000 spectrophotometer (Thermo Fisher Scientific, Waltham, MA, USA) and agarose gel electrophoresis for extracted DNA. The library was constructed using Illumina’s TruSeq Nano DNA LT Library Prep Kit. The end repair process starts by using the End Repair Mix2 in the kit to excise the base protruding from the 5’ end of the DNA, fill in the missing base at the 3’ end, and add a phosphate group to the 5’ end. A separate A-base is then added to the 3’ end of the DNA to prevent self-association of the DNA fragment. PCR amplification of the bacterial 16S rRNA genes V3–V4 region was performed using the forward primer 338F (5’–ACTCCTACGGGAGGCAGCA–3’) and the reverse primer 806R (5’–GGACTACHVGGGTWTCTAAT–3’) (Claesson et al., 2009). Sample-specific 7-bp barcodes were incorporated into the primers for multiplex sequencing. The PCR components contained: 5 × reaction buffer 5 μL, 5 × GC buffer 5 μL, dNTP (2.5 mM) 2 μL, Forwardprimer (10 uM) 1 μL, Reverseprimer (10 uM) 1 μL, DNA Template 2 μL, ddH_2_O 8.75 μL, Q5 DNA Polymerase 0.25 μL. The amplification parameters were initial denaturation 98°C 2 min, followed by 25-30 cycles consisting of denaturation 98°C 15 s, annealing 55°C 30 s, extension 72°C 30 s, final extension 72°C 5 min, 10°C hold. Vazyme VAHTSTM DNA Clean Beads (Vazyme, Nanjing, China) were used to purify PCR amplicons, and the Quant-iT PicoGreen dsDNA Assay Kit was used to quantify them (Invitrogen, Carlsbad, CA, USA). Pair-end 250 bp sequencing was carried out at Shanghai Personal Biotechnology Co., Ltd. using the Illlumina NovaSeq platform and NovaSeq 6000 SP Reagent Kit (500 cycles).

### Data analytics

Amplicons were pooled in equal amounts following the individual quantification step. Taxonomy was assigned to amplicon sequence variants (ASVs) using the classify-sklearn naive Bayes taxonomy classifier in feature-classifier plugin against the silva_132. The NCBI database SRA accession number for of the raw high-throughput sequencing data of leaf bacterium is PRJNA924105. The primer fragments of the sequences were first excised by calling qiime cutadapt trim-paired and the unmatched primer sequences were discarded; then DADA2 was called by qiime dada2 denoise–paired for quality control, denoising, splicing and chimera removal. The above steps were analyzed separately for each library (Callahan et al., 2016). After the denoising of all libraries was completed, the ASVs feature sequences and ASV tables were merged and the total number of denoised sequences (effective sequence volume) was 1,318,062 with an average of 73,225.67 per sample. After removing the chimeras, the total amount of high-quality sequences obtained was 1,029,576 with a mean value of 57,198.67 (49,640 - 63,956).

Venn diagram and histogram of sample ASVs numbers were made with jvenn which is a plug-in for the jQuery Javascript library (Bardou et al., 2014). In order to provide a more com-prehensive assessment of the alpha diversity of the microbial community, the Chao1 and Observed species indices were used to characterize richness (Chao, 1984), the Shannon and Simpson indices to characterize diversity and the Pielou’s evenness index to characterize evenness (Shannon, 1948; Simpson, 1949; Pielou, 1966). Box line plots were created using QIIME2 (2019.4) and the ggplot2 package for the R package (v3.2.0) (Wickham, 2009) and the significance of the differences was verified by Kruskal-Wallis rank sum test and dunn’test as a *post hoc* test. Cluster analysis was performed using the uclust function of the R package (v3.2.0) stat package, using the UPGMA algorithm by default for the Bray–Curtis distance matrix (i.e., the clustering method is average), and visualized using the R package (v3.2.0) script ggtree package (Ramette, 2007). The QIIME2 (2019.4) “qiime taxa barplot” was called to visualize the compositional distribution of each sample at five taxonomic levels: phylum, class, order, family and genus, by counting the feature list after removing singleton. The heatmap was drawn using the pheatmap package in R (v3.2.0) (Gu and Hübschmann, 2022). One-way ANOVA was used to analyze the significant difference of leave chemical properties, and S-N-K q test was used to conduct post-test. Data of significant differences in leave chemical properties were processed by Excel (2019) and analyzed by IBM SPSS 26.0 (Chicago, USA). The linkages between leaf nutrient factors and phyllosphere bacterial community composition and diversity were performed by Redundancy analysis (RDA) *via* Canoco 5. Phylogenetic Investigation of Communities by Reconstruction of Unobserved States (PICRUSt2) took 16S rRNA gene sequences for metabolic pathway prediction in the MetaCyc functional database (https://metacyc.org/) (Douglas et al., 2020). The data were normalized using the sum of the abundances of the EC’s for each sample in parts per million. The average abundance of the second level pathway was calculated using R based on the selected samples.

## Results

### Composition and structure of phyllosphere bacterial communities of different *Populus* spp.

**Figure 1:**
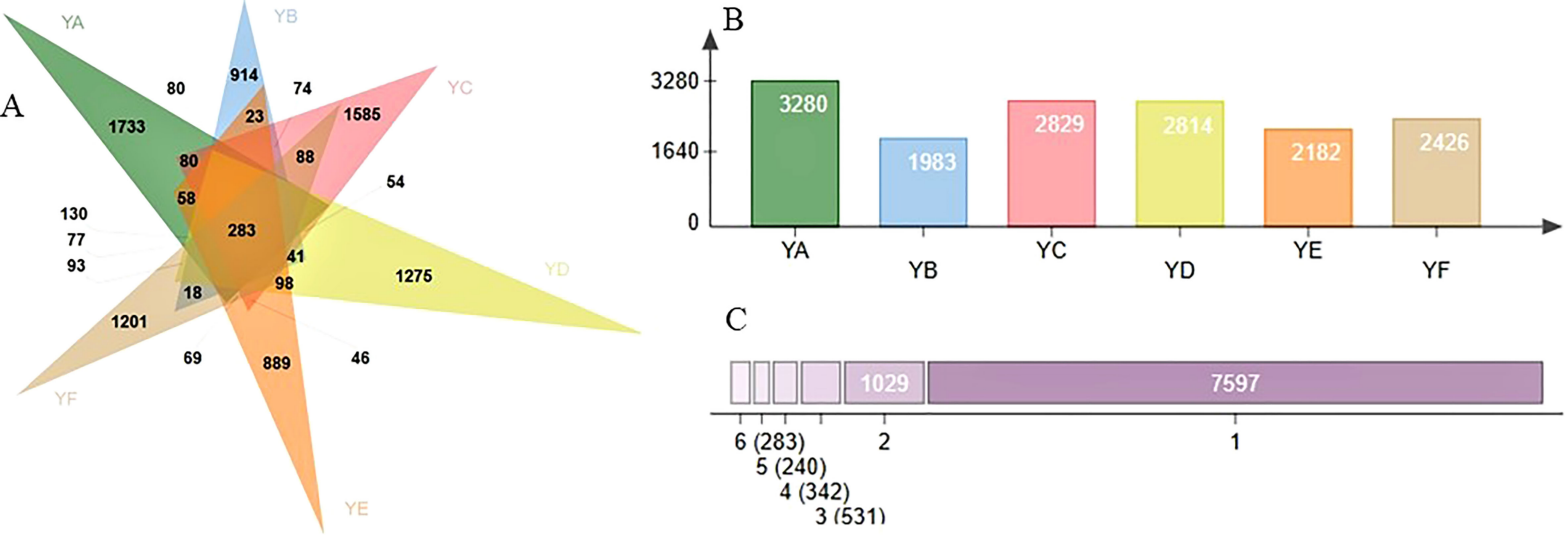
**(A)** venn diagram for different sample leaves; **(B)** number of ASVs per grouping; **(C)** number of ASVs, specific (1) or shared by 2,3, ..., 6. YA: *Populus* × euramaricana ‘Bofeng 3 hao’; YB: P. deltoides ‘Shanghaiguan’ × P. deltides ‘Harvard’; YC: P. nigra ‘N46’; YD: P. nigra ‘N102’; YE: P. × euramericana ‘Guariento’; YF: P. alba × P. glandulosa ‘84k’.

Venn diagrams were produced using the ASV abundance table, as shown in **Figure 1A**. Six species of *Populus* spp. shared 283 ASVs. The number of ASVs was 3280, 1983, 2829, 2814, 2182 and 2426 for YA, YB, YC, YD, YE, and YF, respectively (**Figures 1B, 1C**). The number of ASVs specific to YA (1733) was the highest and YE (889) the lowest (**Figure 1A**).

**Figure 2:**
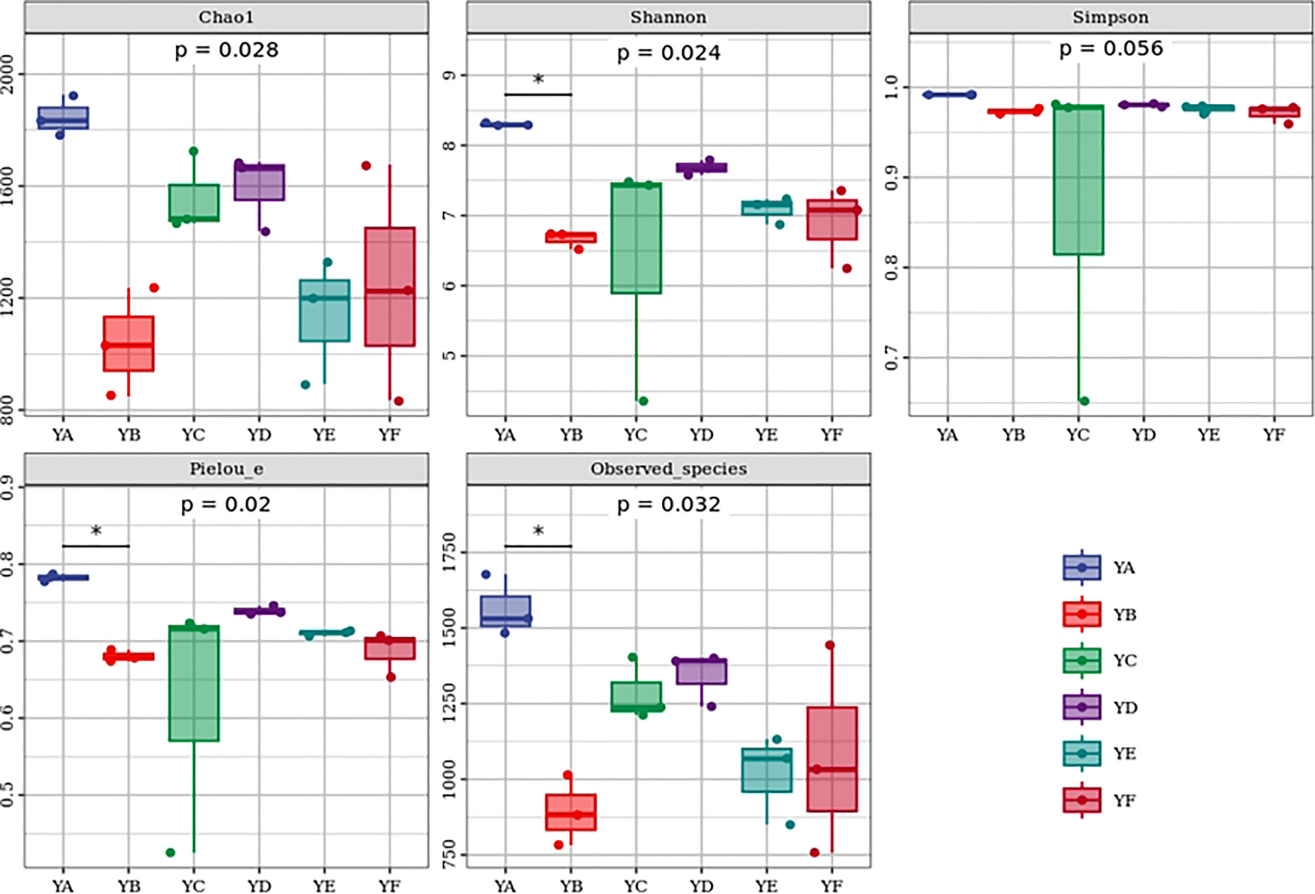
Box plot of alpha diversity of phyllosphere bacterial communities of different species of *Populus* spp.* indicated *p < 0.05* by dunn’test as a post-hoc test. YA: *Populus* × *euramaricana* ‘Bofeng 3 hao’; YB: *P.deltoides* ‘Shanghaiguan’ × *P. deltides* ‘Harvard’; YC: *P.nigra* ‘N46’: YD: *P.nigra* ‘N102’; YE: *P.* × *euramericana* ‘Guariento’; YF: *P. alba* × *P.glandulosa* ‘84k’.

The alpha diversity indices of six different species of *Populus* spp. were analyzed based on the Kruskal-Wallis algorithm and differences were found between them. Chao 1, Shannon, Observed_species, Pielou_e indices were significantly different between groups (*p* < 0.05), while Simpson index was not significantly different between groups (*p* > 0.05). YA had the highest Chao 1, Shannon, Simpson, Observed_species, Pielou_e indices. The lowest mean values of Chao1 and Observed_species indices were found in YB, and the lowest mean values of Shannon, Simpson, Pielou_e indices were found in YC (**Figure 2**).

**Figure 3:**
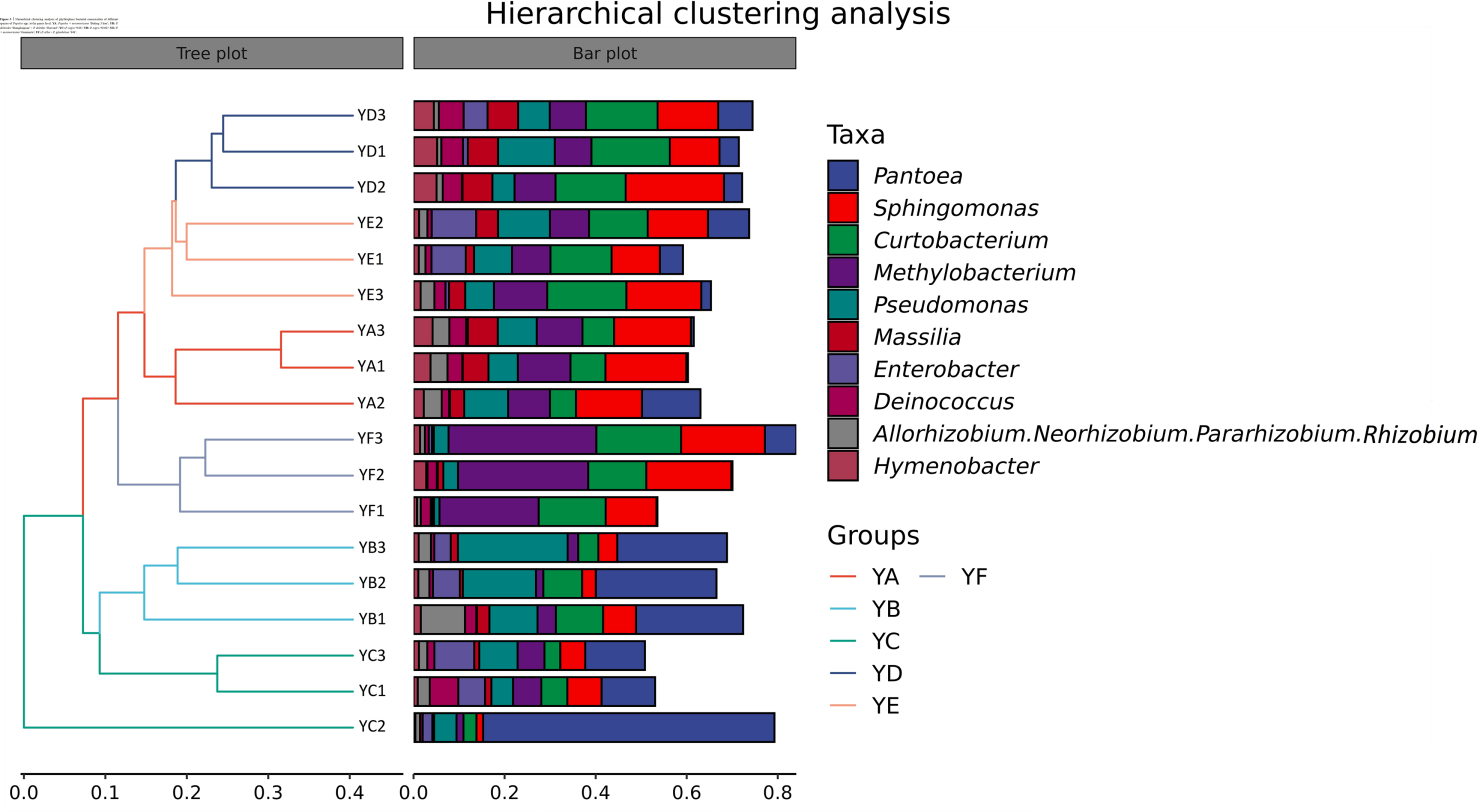
Hierarchical clustering analysis of phyllosphere bacterial communities of different species of *Populus* spp. at the genus level. YA: *Populus* x *euramaricana* 'Bofeng 3 hao'; YB: *P. deltoides* 'Shanghaiguan' *P. deltides* 'Harvard'; YC: *P. nigra* 'N46'; YD: *P. nigra 'N102'*; YE: *P.* x *euramericana* 'Guariento'; YF: *P. alba* × *P. glandulosa* '84k'.

**Figure 4:**
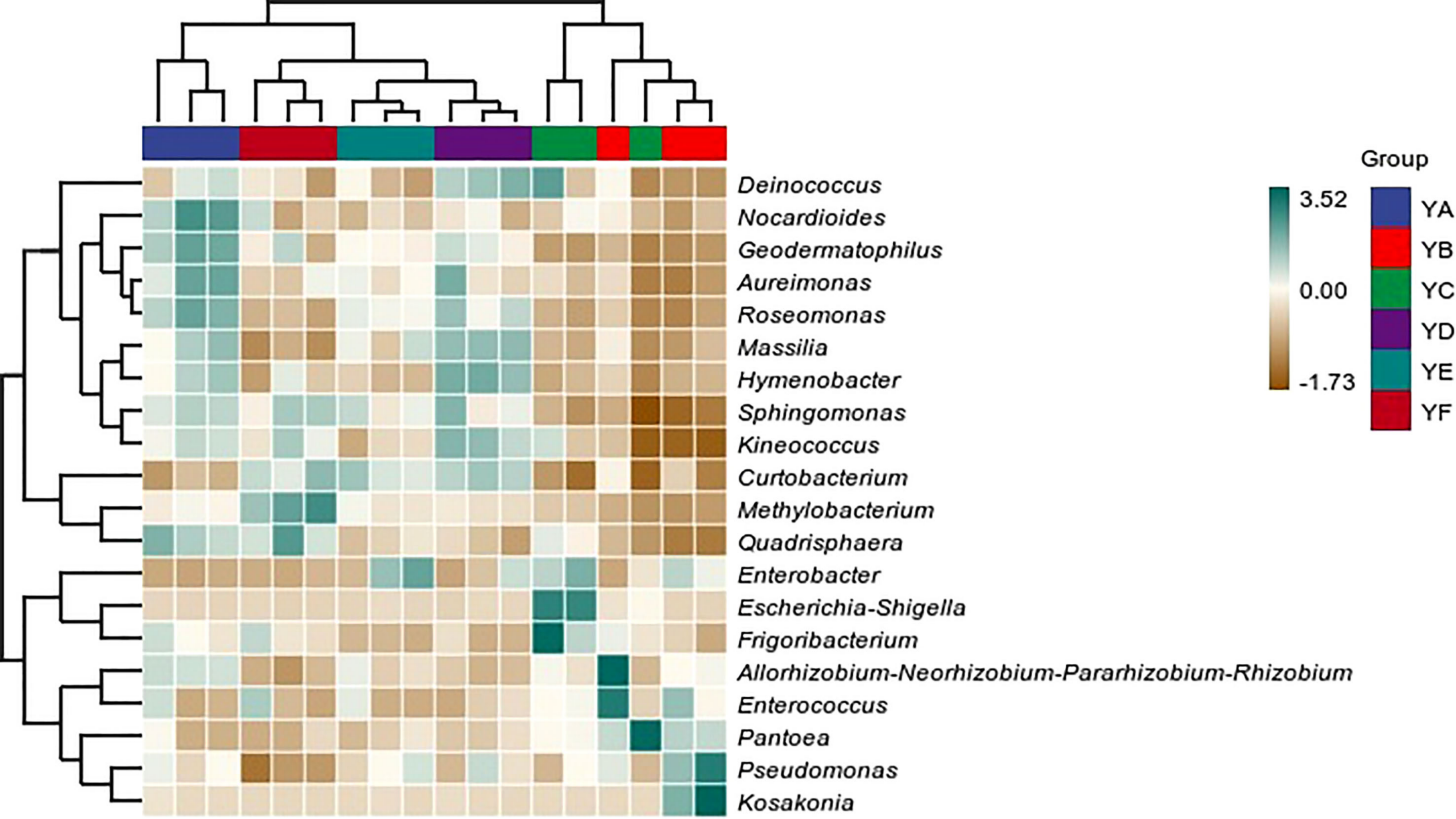
Heatmap of phyllosphere bacterial communities of different species of *Populus* spp. at the genus level. YA: *Populus* x *euramaricana* 'Bofeng 3 hao'; YB: *P. deltoides* 'Shanghaiguan' × *P. deltides* 'Harvard'; YC: *P. nigra* 'N46'; YD: *P. nigra* 'N102'; YE: *P.* x *euramericana* 'Guariento'; YF: *P. alba* × *P. glandulosa* '84k'.

Hierarchical clustering analysis of phyllosphere bacterial communities at the genus level using Bray-Curtis distance algorithm and average clustering was performed as in **Figure 3**. YB and YC were clustered into one category alone and the remaining four treatments were clustered into one category. YD was more similar to YE. The histogram on the right indicates that the microbial communities were not equally abundant at the genus level, and although the species composition of the treatments was similar, there were differences in abundance. The heatmap also confirmed this conclusion (**Figure 4**).

Statistical analysis of the sampled ASVs resulted in a table of the specific composition of phyllosphere bacteria in each sample for each taxonomic level. With this table, it was possible to calculate the composition of taxonomic units contained in each taxonomic level for each of the six samples. Statistically, high-throughput sequencing yielded a total of 31 phyla, 76 classes, 179 orders, 313 families, 683 genera and 940 species.

**Figure 5:**
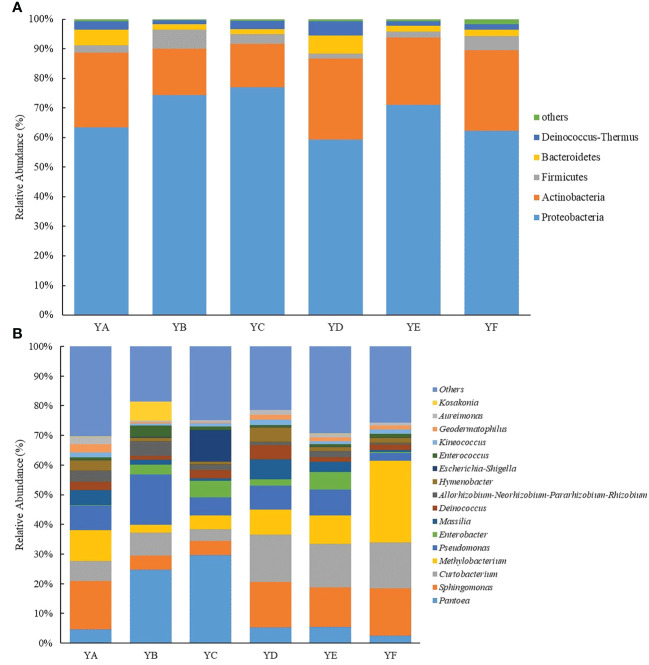
Analysis of the composition of phyllosphere bacterial communities in different *Populus* spp. at all levels of taxonomic units. **(A)** at the phylum level; **(B)** at the genus level; YA: *Populus* x *euramaricana* 'Bofeng 3 hao'; YB: *P. deltoides* 'Shanghaiguan' *P. deltides* 'Harvard'; YC: *P. nigra* 'N46'; YD: *P. nigra* 'N102'; YE: *P.* x *euramericana* 'Guariento: YF: *P. alba* × *P. glandulosa* '84k'.

At the phylum level, the relative abundance of phyllosphere bacteria greater than 1% were Proteobacteria, Actinobacteria, Firmicutes, Bacteroidetes, Deinococcus-Thermus. Among them, Proteobacteria and Actinobacteria were the dominant phylum with relative abundance greater than 10%. The highest relative abundance content of Proteobacteria was found in YC (76.96%) and the lowest in YD (59.37%). Conversely, Actinobacteria had the highest relative abundance in YD (27.31%) and the lowest in YC (14.69%). Firmicutes had the significantly highest relative abundance in YB at 6.43%. Bacteroidetes had higher relative abundance in YD and YA at 6.20% and 5.24%, respectively (**Figure 5A**).

The top ten phyllosphere bacterial classes in relative abundance were Gammaproteobacteria, Alphaproteobacteria, Actinobacteria, Bacilli, Bacteroidia, Deinococci, Deltaproteobacteria, Saccharimonadia, Thermoleophilia, Acidimicrobiia. Gammaproteobacteria had the highest relative abundance among the six samples at 61.75%, while Alphaproteobacteria (14.98%) and Actinobacteria (14.12%) had the lowest relative abundance among the six samples. Alphaproteobacteria had the highest relative abundance in YF (49.33%) and Actinobacteria had the highest abundance in YD (26.97%) (**Supplementary Figure 2A**). At the order level, Enterobacteriales had the highest abundance in YC at 53.29%, while YA contained only 7.05%. Micrococcales varied little in abundance across the six *Populus* spp. leaves, ranging from 9.88% (YC) to 20.44% (YD). Rhizobiales had the highest abundance in YF at 30.63% and only 9.05% in YB (**Supplementary Figure 2B**). At the family level, the relative abundance of Enterobacteriaceae was as high as 53.29% in YC and less than 10% in both YA and YF. Microbacteriaceae had the highest abundance in YD (19.93%), Sphingomonadaceae had the highest abundance in YA (17.72%), and Beijerinckiaceae had the highest abundance in YF (28.04%) (**Supplementary Figure 2C**).

At the genus level, the genera ranked greater than 1% in relative abundance were *Pantoea*, *Sphingomonas*, *Curtobacterium*, *Methylobacterium*, *Pseudomonas*, *Massilia*, *Enterobacter*, *Deinococcus*, *Allorhizobium-Neorhizobium-Pararhizobium-Rhizobium*, *Hymenobacter*, *Escherichia-Shigella*, *Enterococcus*, *Kineococcus*, *Geodermatophilus*, *Aureimonas*, *Kosakonia*. *Pantoea* was the most abundant in YC with 29.62%, while in YF it was only 2.45%. the relative abundance of *Sphingomonas* in YA, YD, YE, YF was greater than 10%. *Curtobacterium* had the highest relative abundance in YD (16.12%) and *Methylobacterium* the highest in YF (27.62%). 2.55% of *Pseudomonas* was found in YF but 16.91% in YB (**Figure 5B**).

### Variation in leaf nutrient indices among six *Populus* spp.

The six species of *Populus* spp. varied in their basal nutrient indexes in leaves. While the differences in soluble sugar and starch content were not statistically significant (*p* > 0.05), there were significant differences in their carbon, nitrogen, phosphorus, and carbon/nitrogen ratios (*p* < 0.05). YC had the lowest carbon/nitrogen ratio (average value of 14.16), the highest N content (average value of 31.37 g/kg), and the highest phosphorus content (average value of 6.40 g/kg). The highest carbon/nitrogen ratio (mean value 17.79) and highest average carbon content (mean value 467.34 g/kg) were both found in the YF. YA had the least amount of soluble sugar, starch, phosphorus, nitrogen, and carbon (**Table 1**).

**Table 1:** Variation in the chemical properties of the leaves of different species of *Populus* spp.

	Carbon/g kg^-1^	Nitrogen/g kg^-1^	Carbon/nitrogen	Phosphorus/g kg^-1^	Soluble sugar/mg kg^-1^	Starch/mg kg^-1^
YA	439.26 ± 5.47 B	25.19 ± 0.97 C	17.51 ± 0.64 A	2.09 ± 0.16 C	15.72 ± 3.76 A	57.52 ± 4.61 A
YB	457.55 ± 9.45 AB	30.03 ± 1.46 AB	15.30 ± 0.45 BC	3.47 ± 0.41 B	21.93 ± 1.51 A	60.76 ± 2.16 A
YC	443.82 ± 0.94 B	31.37 ± 0.56 A	14.16 ± 0.27 C	6.40 ± 0.41 A	24.08 ± 5.53 A	74.13 ± 6.77 A
YD	442.33 ± 7.68 B	27.10 ± 0.33 BC	16.33 ± 0.20 AB	2.51 ± 0.02 C	22.91 ± 0.93 A	71.73 ± 1.94 A
YE	450.98 ± 3.71 AB	30.31 ± 0.26 AB	14.88 ± 0.08 BC	2.52 ± 0.06 C	26.27 ± 2.25 A	68.80 ± 6.75 A
YF	467.34 ± 1.75 A	26.46 ± 1.33 C	17.79 ± 0.87 A	2.10 ± 0.06 C	21.61 ± 2.89 A	68.44 ± 4.50 A
F	3.493	6.974	8.635	44.914	1.233	1.759
*p*	0.022	0.001	<0.001	<0.001	0.335	0.172

Data were average ± standard error. Different capital letters meant significant difference at 0.05 level. YA: *Populus* × euramaricana ‘Bofeng 3 hao’; YB: P. deltoides ‘Shanghaiguan’ × P. deltides ‘Harvard’; YC: P. nigra ‘N46’; YD: P. nigra ‘N102’; YE: P. × euramericana ‘Guariento’; YF: P. alba × P. glandulosa ‘84k’.

### Effect of leaf nutrient factors on the composition and structure of phyllosphere bacterial communities

**Figure 6:**
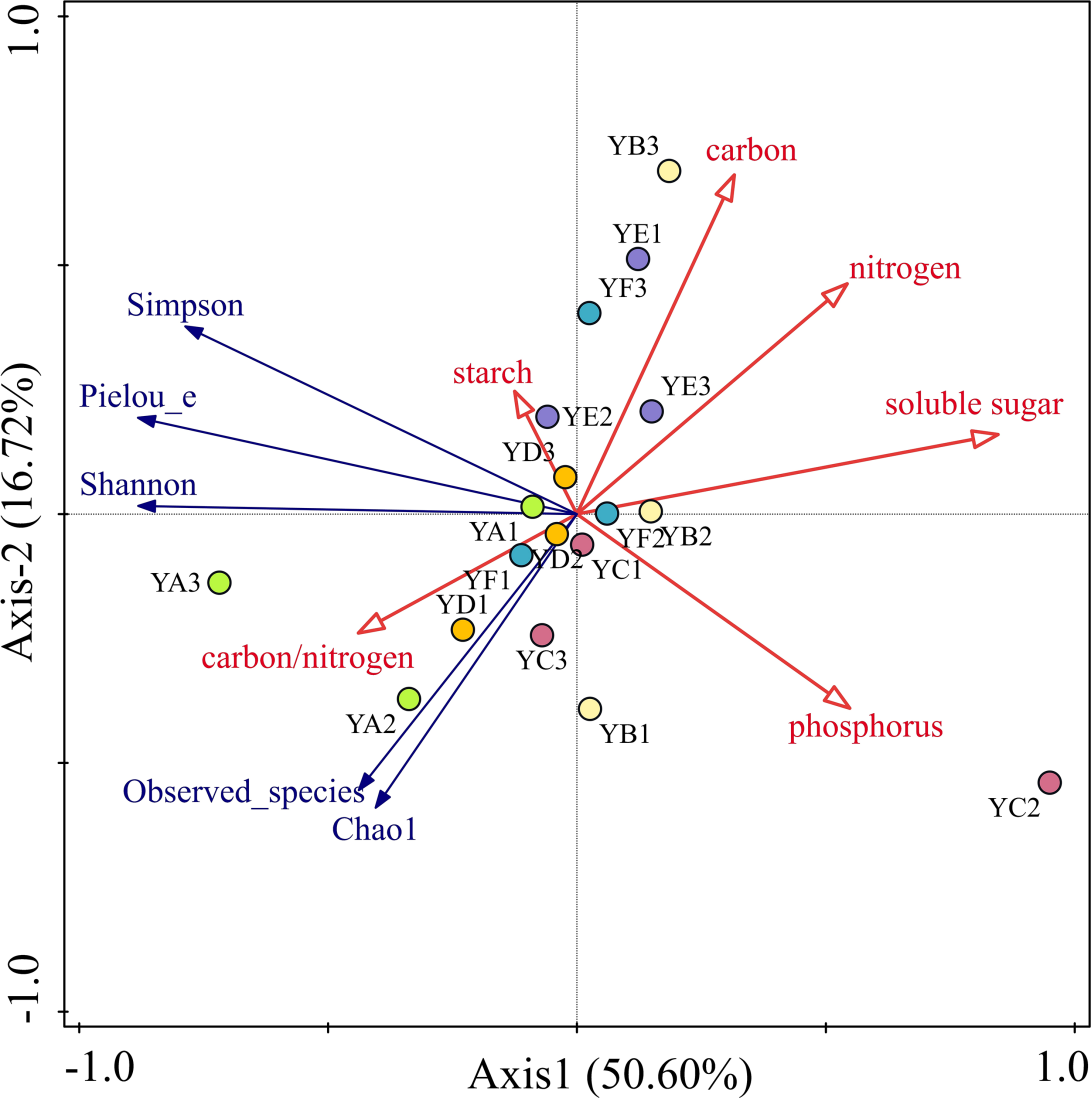
Redundancy analysis (RDA) of leaf nutrient factors and alpha diversity of phyllosphere bacterial communities. YA: *Populus* x *euramaricana* 'Bofeng 3 hao'; YB: *P. deltoides* 'Shanghaiguan' *P. deltides* 'Harvard'; YC: *P. nigra* 'N46'; YD: *P. nigra* 'N102'; YE: *P.* x *euramericana* 'Guariento'; YF: *P. alba P.*× *P. glandulosa* '84k'.

To better understand the relationship between phyllosphere bacterial communities diversity and leaf nutrient factors, Detrended correspondence analysis (DCA) was carried out on the alpha diversity and soil chemical properties of phyllosphere bacterial communities, and the result showed that the maximum gradient length = 0.10 < 3, indicating that the distribution of different species of poplar phyllosphere bacterial communities was closer to the linear model and that the redundancy analysis (RDA) could better explain the relationship between them. The RDA indicated that six leaf nutrient factors explained a total of 67.53% of the overall eigenvalues and had significant effects on phyllosphere bacterial community diversity. The eigenvalues of the first two sorting axes of the RDA explained 50.6% and 16.72% of the variation in phyllosphere bacterial communities’ diversity, respectively (**Figure 6**). And only soluble sugar (*p* =0.004), and starch (*p* =0.046) among the six nutrient factors had a significant effect on phyllosphere bacterial community diversity, explaining 54.3%, and 17.6% of the variation in phyllosphere bacterial community diversity. Carbon/nitrogen was positively correlated with all five indices and the starch content was only positively correlated with the Simpson, Pielou_e and Shannon indices. The remaining leaf nutrient factors all had a negative effect on the alpha diversity index.

DCA of the relative abundance of phyllosphere bacterial communities at the phylum level and genus level greater than 1% with leaf nutrient factors showed that the maximum gradient length = 0.36 (phylum), 0.6 (genus), both less than 3. This indicates that RDA could better explain the relationship between them.

**Figure 7:**
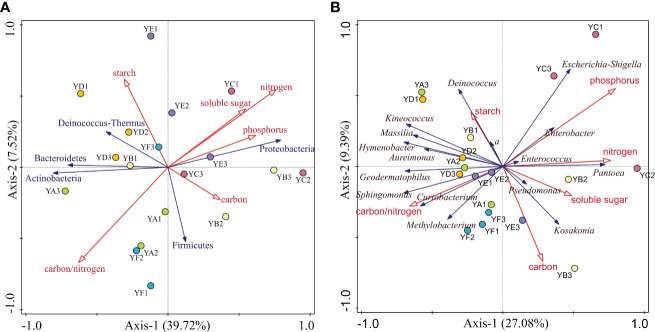
Redundancy analysis (RDA) of leaf nutrient factors and relative abundance > 1% of phyllosphere bacterial communities at the phylum **(A)** and genus **(B)** level. YA: *Populus* x *euramaricana* 'Bofeng 3 hao'; YB: *P. deltoides* 'Shanghaiguan' x *P. deltides* 'Harvard'; YC: *P. nigra* 'N46'; YD: *P. nigra* 'N102'; YE: *P.* x *euramericana* 'Guariento'; YF: *P. alba* × *P. glandulosa* '84k'.

At the phylum level, RDA showed that the six leaf nutrient factors together explained 53.58% of the overall eigenvalues (**Figure 7A**). The eigenvalues from the first two ordination axes of RDA explained 39.72% and 7.52% of the variation in phyllosphere bacterial community’s diversity, respectively. Only leaf nitrogen content had a significant effect on the phyllosphere bacterial clade, explaining 47.1% of the variation in community diversity (*p* = 0.008). At the genus level, the eigenvalues from the first two ordination axes of RDA explained 27.08% and 9.39% of the variation in phyllosphere bacterial community’s diversity (**Figure 7B**). Only 48.8% of the eigenvalues are explained on the four axes. Leaf phosphorus content had a significant effect on the phyllosphere bacterial community diversity (*p* = 0.002).

### Prediction of the functional potential of phyllosphere bacterial communities of different *Populus* spp.

**Figure 8:**
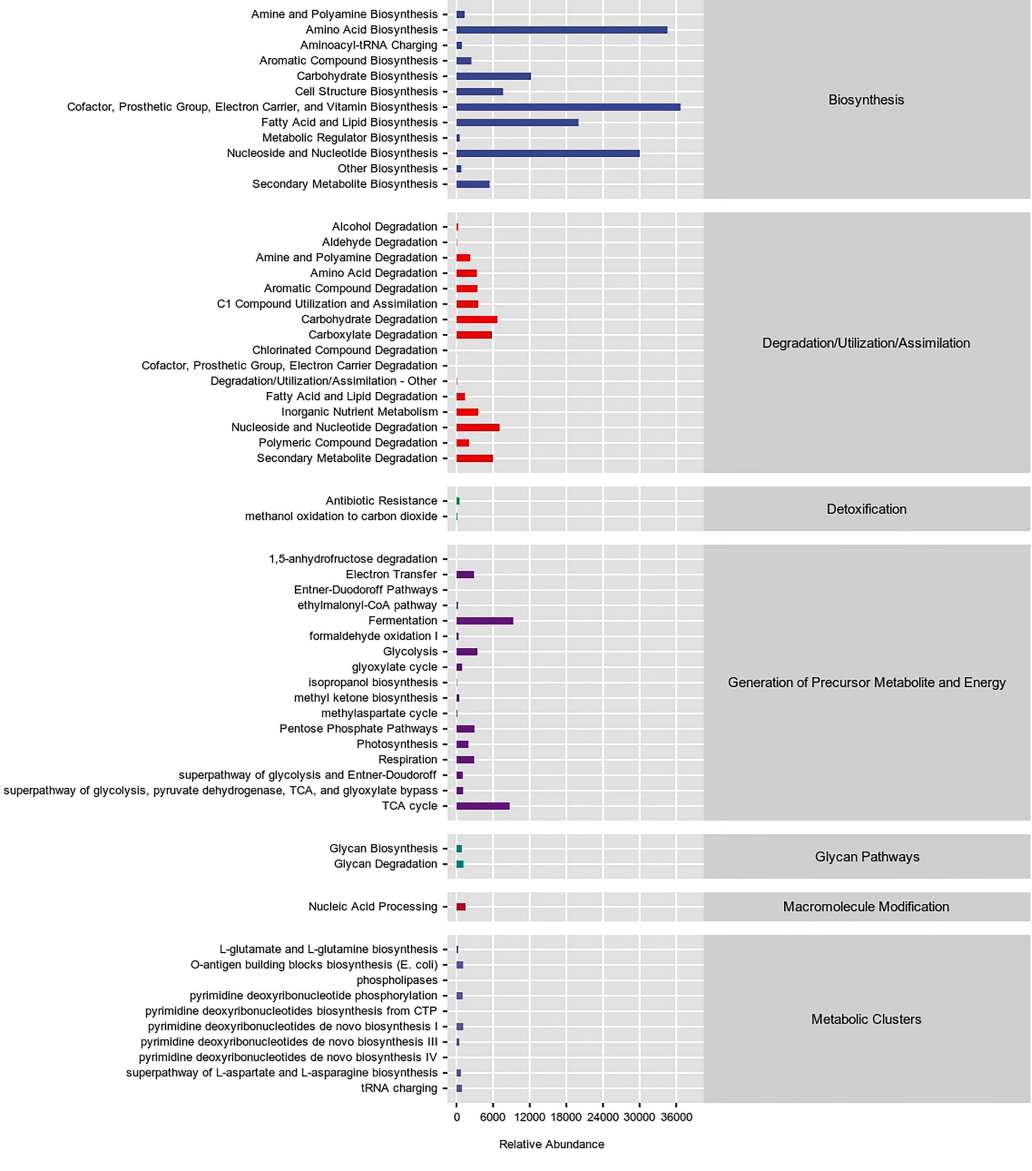
Predicting metabolic pathway statistics in interleaf bacterial communities based on the MetaCyc database.

The analysis of the bacterial community based on the MetaCyc database (**Figure 8**) showed that at the primary level, there were Biosymthesis, Degradation/Utilization/Assimilation, Detoxification, Generation of Precursor Metabolite and Energy, Glycan Pathways, Macromolecule Modification, and Metabolic Clusters. The highest relative abundance of bacteria was associated with Biosynthesis at 62.47%.

The top three metabolic functions studied at the secondary level were Cofactor, Prosthetic Group, Electron Carrier, and Vitamin Biosynthesis (15.03%), Amino Acid Biosynthesis (14.15%) and Nucleoside and Nucleotide Biosynthesis (12.29%). The flora also had metabolic functions, for example, Fatty Acid and Lipid Biosynthesis (8.19%), Carbohydrate Biosynthesis (5.00%), Fermentation (3.81%), TCA cycle (3.56%), Cell Structure Biosynthesis (3.13%), Nucleoside and Nucleotide Degradation (2.89%), Carbohydrate Degradation (2.74%), Secondary Metabolite Degradation (2.45%), Carboxylate Degradation (2.38%), Secondary Metabolite Biosynthesis (2.22%), C1 Compound Utilization and Assimilation (1.45%), Inorganic Nutrient Metabolism (1.45%), Glycolysis (1.40%), Aromatic Compound Degradation (1.40%), Amino Acid Degradation (1.37%), Pentose Phosphate Pathways (1.20%), Respiration (1.18%), Electron Transfer (1.18%), Aromatic Compound Biosynthesis (1.00%). Other than that, there were 38 metabolic functions with a relative abundance of less than 1%.

## Discussions

The present study found differences in the composition and structure of the phyllosphere bacterial community of different *Populus* spp. under the same off-site conditions, which answers the first hypothesis presented in the previous section. A large proportion of the variation in the composition of phyllosphere microbial communities is explained by the hosts themselves (Laforest-Lapointe et al., 2016). Plant genotypes have a decisive influence on the composition of phyllosphere microbial communities (Whipps et al., 2008; Bálint et al., 2013; Bodenhausen et al., 2014). Current studies have shown that phyllosphere microbial community structure differs significantly between plants, that phyllosphere microbes of the same species vary with their geographical location, and that the community composition of phyllosphere microbes is distinctly host-specific (Finkel et al., 2012; Rastogi et al., 2012). Finkel et al. (2011) also found that geographical location, rather than tree species, was the main determinant of bacterial communities in the phyllosphere. The six *Populus* spp. in this study were all harvested from the same habitat and were similar in elevation, temperature, rainfall and sunlight, which may explain the differences in phyllosphere microbial community composition between them, but the alpha diversity indices were less significantly different (*p*<0.05).

A correlation heat map analysis of physicochemical properties and alpha diversity of the phyllosphere bacterial community and the top ten phyla and genera in relative abundance revealed that leaf carbon and soluble sugar content were the main and negative influences on the alpha diversity index. The bacterial composition of the phyllosphere was mainly influenced by leaf nitrogen content, carbon/nitrogen, at the phylum and genus level. Leaf phosphorus content also significantly influenced the relative abundance of phyllosphere bacterial genera. Previous studies have shown that the growth of interleaf bacteria is limited mainly by carbon and to a lesser extent by nitrogen (Delmotte et al., 2009; Kembel and Mueller, 2014), but in the present study the effect of leaf carbon content on bacteria was not significant. These essential nutrients are available resources for bacteria living on the leaf surface and thus influence the community structure of phyllosphere bacteria (Gong and Xin, 2021). Kembel et al. (2014) found that leaf nitrogen and phosphorus content effected the phyllosphere bacterial community. Yadav et al. (2005) demonstrated that leaf shape, marginal folds and stomata as well as leaf chemistry (nitrogen, phosphorus, soluble carbohydrates and water content) affect the community composition of phyllosphere microorganisms, resulting in different interleaf bacterial colonization. These previous studies all support the conclusions reached in this study.

The dominant taxa of phyllosphere bacteria are mainly Proteobacteria and Actinobacteria, which is consistent with the findings of other scholars (Delmotte et al., 2009; Rastogi et al., 2012). Additionally, there were also significant concentrations of the relatively abundant phyla Firmicutes, Bacteroidetes, Deinococcus-Thermus, Patescibacteria, Acidobacteria, Gemmatimonadetes, Chloroflexi, and FBP. Previous studies have shown that the bacterial community phylogenetic structure of the phyllosphere microbial community contains relatively few phyla, dominated by Proteobacteria, Actinobacteria, Firmicutes and Bacteroidetes, which generally dominate the bacterial community composition (Redford et al., 2010; Kembel et al., 2014; Kecskeméti et al., 2016; Grady et al., 2019). Furthermore, microflora belonging to the Proteobacteria phylum are rich in metabolic diversity and have a variety of functions in the interleaf bacterial community such as methylotrophy, nitrification, nitrogen fixation and non-oxygenic photosynthesis (Fürnkranz et al., 2008; Atamna-Ismaeel et al., 2012; Watanabe et al., 2016). Besides Proteobacteria, microorganisms of the Bacteroidetes and Actinobacteria all perform different ecological functions in the phyllosphere environment (Romero et al., 2016). A number of major bacterial genera, including *Methylobacterium*, *Pseudomonas*, *Bacillus*, *Massilia*, *Sphingomonas*, *Arthrobacter* and *Pantoea*, appear to constitute the core phyllosphere microbial taxa (Rausch et al., 2001; Lopez-Velasco et al., 2011; Knief et al., 2012; Rastogi et al., 2012). The relative abundance of the genera *Pantoea*, *Sphingomonas*, *Curtobacterium*, *Methylobacterium*, were found to be high in *Populus* spp. phyllosphere bacteria. The interactions of some phyllosphere microbial communities provide some protection to plants (Innerebner et al., 2011; Ottesen et al., 2015). For example, bioactive molecules produced by Pseudomonas strains (e.g., Coronatine and Syringolin A) can induce stomatal closure and thus affect the entry of pathogens into apoplast (Melotto et al., 2006; Hunter et al., 2010). The phyllosphere microbes are also able to cycle carbon and nitrogen through the direct use of carbohydrates released by plants or secreted by arthropods, the interception of ammonium atmospheric pollutants by nitrifying bacteria and nitrogen fixation (Müller and Ruppel, 2014).

In summary, in our study it was found that different *Populus* spp. under the same stand conditions resulted in different phyllosphere bacterial communities. While bacterial community structure was mainly influenced by leaf carbon and soluble sugar content, leaf nitrogen, phosphorus and carbon/nitrogen were the main factors affecting the relative abundance of phyllosphere bacteria. This provides theoretical support for the study of the composition and structure of phyllosphere bacteria in woody plants and the factors influencing them.

## Data availability statement

The datasets presented in this study can be found in online repositories. The names of the repository/repositories and accession number(s) can be found below: NCBI, accession number PRJNA924105.

## Author contributions

CD and WenZ designed the research. JL, and WeiZ performed the research. JL, YL, ZY and WeiZ analyzed the data. WeiZ, JL, WenZ and CD wrote the manuscript. Project administration, XS and CD. Funding acquisition, XS and CD. All authors contributed to the article and approved the submitted version.
