# Supplementary material for: Differences in phyllosphere microbiomes among different Populus spp. in the same habitat
Source: Front Plant Sci. 2023 Mar 30;14:1143878. doi: 10.3389/fpls.2023.1143878 (PMC10098339; doi:10.3389/fpls.2023.1143878)
Supplement: Supplementary file 1 [file DataSheet_1.docx]

Supplementary Material


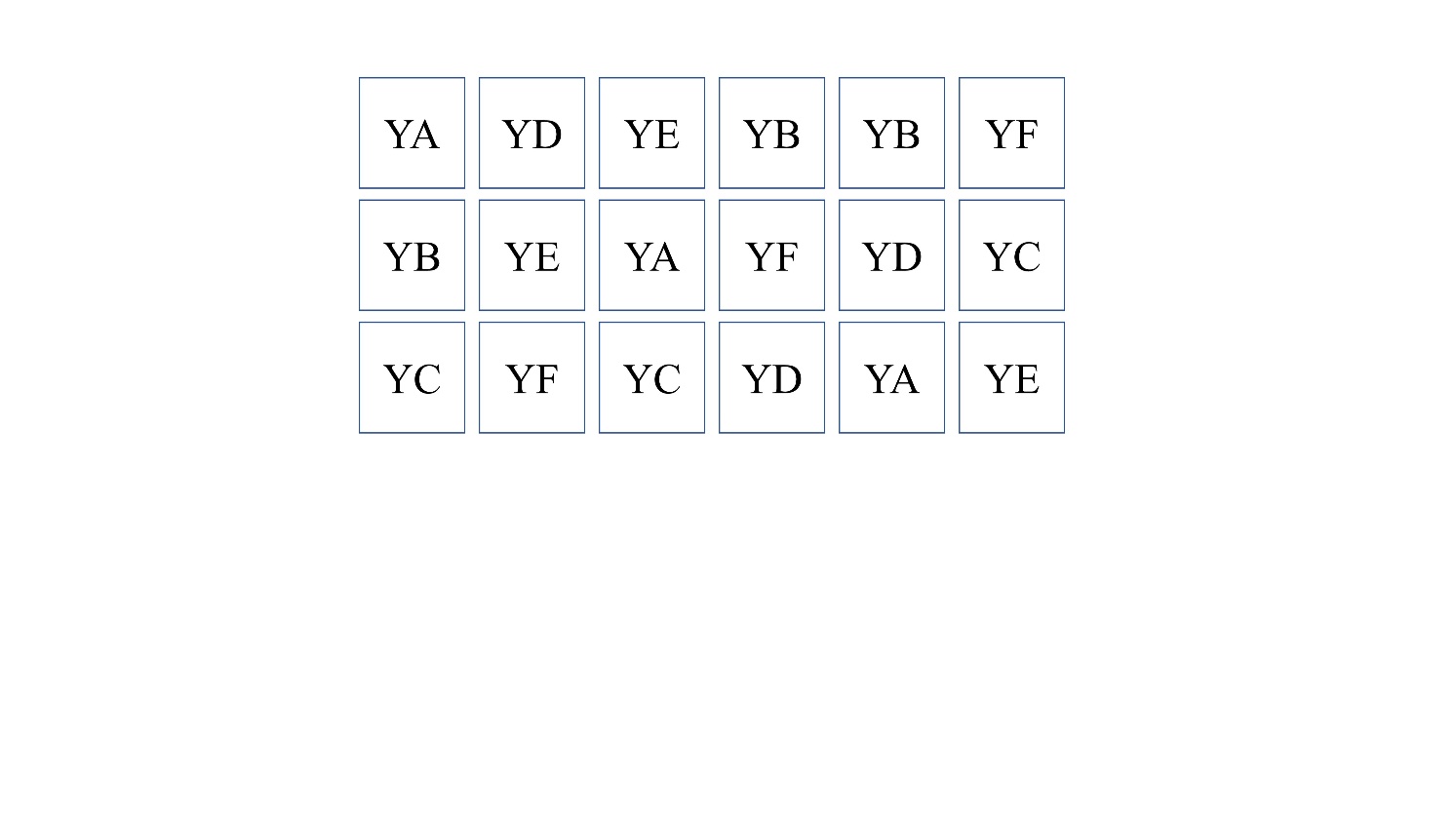


**Supplementary Figure 1.** Site information. **YA**: *Populus × euramaricana* ‘Bofeng 3 hao’; **YB:** *P. deltoides* ‘Shanghaiguan’ × *P. deltides* ‘Harvard’; **YC:** *P. nigra* ‘N46’; **YD:** *P. nigra* ‘N102’; **YE:** *P. × euramericana* ‘Guariento’; **YF:** *P. alba* × *P. glandulosa* ‘84k’


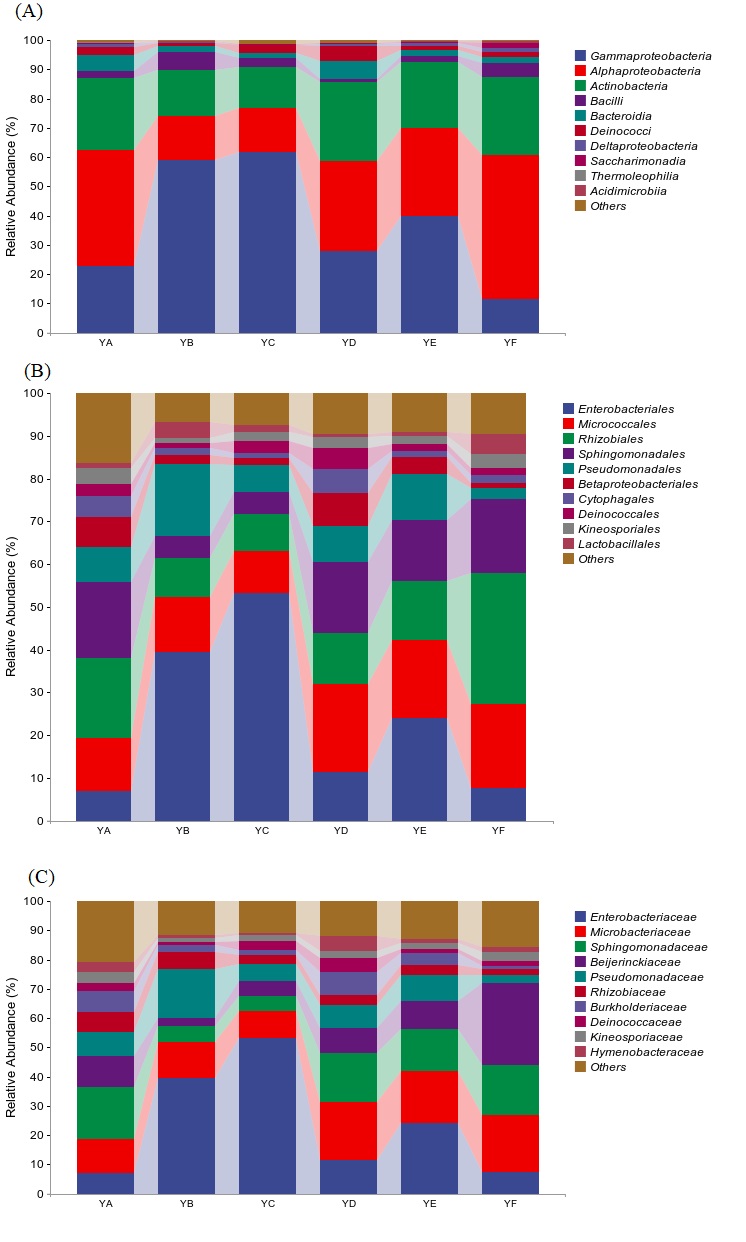


**Supplementary Figure 2.** Analysis of the composition of phyllosphere bacterial communities in different *Populus* spp. at all levels of taxonomic units. (A): at the class level; (B): at the order level; (C): at the family level; **YA**: *Populus × euramaricana* ‘Bofeng 3 hao’; **YB:** *P. deltoides* ‘Shanghaiguan’ × *P. deltides* ‘Harvard’; **YC:** *P. nigra* ‘N46’; **YD:** *P. nigra* ‘N102’; **YE:** *P. × euramericana* ‘Guariento’; **YF:** *P. alba* × *P. glandulosa* ‘84k’
